# Reporting on 16 years of laboratory capacity building while exploring the future of WOAH's Laboratory Twinning Programme

**DOI:** 10.3389/fvets.2022.1058335

**Published:** 2022-11-24

**Authors:** Mariana Marrana, Emmanuel Appiah, Morgan Jeannin, William Gilbert, Adriana Nilsson, Keith Hamilton, Jonathan Rushton

**Affiliations:** ^1^World Organisation for Animal Health, Paris, France; ^2^Institute of Infection, Veterinary and Ecological Sciences, School of Health and Life Sciences, University of Liverpool, Liverpool, United Kingdom; ^3^Work, Organisation and Management Group, Management School, University of Liverpool, Liverpool, United Kingdom

**Keywords:** animal health, laboratories, veterinary laboratories, twinning, sustainability, public health

## Abstract

Animal health laboratories are an increasingly important part of safeguarding animal and public health due to their role in surveillance and diagnostics of animal diseases, food safety, and in the development and production of medicinal products, vaccines, and diagnostic tools. Despite their importance, the global distribution of veterinary laboratory expertise is uneven, with greater concentration of reference laboratories in wealthier countries. To address this issue, the World Organization for Animal Health (WOAH, founded as OIE) created a Laboratory Twinning Programme in 2006. The paper will briefly review this Programme in the context of an increasingly populated global health security field, based on a literature review and on a combination of public and internal WOAH data and describe the implementation of the Programme in the past 16 years, noting the drivers for project implementation, its links with the global livestock biomass distribution and with the current distribution of veterinary laboratory expertise. There has been broad uptake and diversity in the focus of the twinning projects implemented in WOAH Member Countries. The Laboratory Twinning Programme would benefit from an evaluation that looks at its outcomes and quantifiable impact in beneficiary countries. A case is made for the development of a monitoring and evaluation system tailored to the Programme's specificities.

## Introduction

Development assistance in the context of global health is an increasingly populous and fragmented field (1). A literature review covering the political economy of foreign aid allocation in the context of global health initiatives led to the conclusion that most aid is unequivocally motivated and still reflects old colonial relationships and western-led geopolitical relationships (2, 3). However, it has not been possible to determine whether aid that is not overtly politically driven, such as that of charities, philanthropies, and non-governmental organizations, is in any way less effective than “official development assistance” allocated by a donor country to a beneficiary country (4). Foreign aid has contributed to remarkable improvements in health, education, and poverty, during the 20th century, thereby spurring economic growth in numerous developing countries (5). Given the socioeconomic advancements experienced by countries that had previously been in the low-middle income level, new donors have arisen in the development assistance space. These include, but are not limited to, the BRICs: Brazil, Russia, India, China, and South Africa. These countries tend to have a more equalitarian relationship between donor and beneficiary, framing development assistance projects as “technical cooperation,” and to allocate foreign aid irrespective of the political system of the recipient– a radical departure from the model used by Western donors, which tend to give more aid to democratizing countries and to frame development assistance as “capacity building” initiatives (2, 6–8). International and intergovernmental organizations have increasingly acted as intermediaries in foreign aid allocation, contributing to the multilateralization of the field of development assistance, especially in relation to global health (9, 10). International organizations working in this field have increased in number and in scope, as have philanthropic organizations, thereby resulting in a densely populated field, which is hard to coordinate and steer, with organizations frequently overstepping each other's mandates (1, 3).

Disease emergence at the interface between animals and humans has always been important, but is becoming increasingly more so (11). Given that the majority of infectious diseases are of zoonotic origin, animal health practitioners have a key role to play in preventing, detecting and mitigating the emergence of zoonoses at their source (12). More specifically, animal health laboratories have a critical role to play in the detection of new infectious agents and in supporting surveillance and alerting for the occurrence of new and known diseases in their geographic area. These laboratories have a broad range of competencies, from food safety to diagnostics, vaccine production and quality control, research and development, etc., ultimately impacting public health along with animal health. The capacity, reach, and resources of national veterinary services vary widely across countries, which is reflected in the global distribution of veterinary laboratory expertise (13). WOAH Reference Centers are laboratories and other types of scientific institutes which have the capacity to uphold the standards of the WOAH *Terrestrial* and *Aquatic Manual of Diagnostic Tests and Vaccines*, and to contribute for scientific progress in their fields through active research and development. As of May 2022, there were 266 WOAH Reference Centers globally. These institutes are expected to support animal health systems globally through testing of samples, to provide training and advice to other Member Countries, and to collaborate with dedicated scientific networks.

Only some developed, high-income, nations have the capacity to systematically assess the health status of their livestock and wildlife populations through surveillance and monitoring programmes and look for pathogens that could spill-over to humans. These, countries benefit from early-warning systems, which allow them to detect outbreaks sooner and to reduce the socioeconomic impact of animal diseases on animal and public health systems. This leaves poorer countries in a position where they must bear a heavier socioeconomic burden caused by animal diseases (14), in part caused by their limitations in implementing surveillance an monitoring systems. However, pathogens travel across borders and oceans through travel, trade, and migrations, and the missed opportunity of early detection results in higher likelihood of international disease spread. Therefore, countries and organizations concerned with global health security provide support to laboratories of developing countries in an effort to improve their capacity for detection and control of diseases, thereby contributing to evening out the global laboratory expertise and technical capacity, including that of animal health laboratories. One such initiative is the World Organization for Animal Health (WOAH, founded as OIE) Laboratory Twinning Programme, which was founded in 2006 with the aim to support the development of veterinary laboratory expertise in underserved regions. In these projects, one WOAH Reference Center is paired with an institute from another WOAH Member Country and together the institutes develop and implement a 2–3-year workplan focused on a disease or on a topical area. The Delegates of the countries and institute directors must confirm their agreement and support to the twinning initiative. There has been broad uptake and diversity in the focus of twinning projects implemented under the scope of this programme. Yet, a formal evaluation of the Programme's impact and the sustainability of the outcomes of individual projects has not been done. In this paper, we describe the implementation of the Programme in the past 16 years, making a connection with the global distribution of livestock biomass and the current distribution of veterinary laboratory expertise. This paper makes a case for the development of a monitoring and evaluation (M&E) tool for WOAH Laboratory Twinning projects which will inform similar capacity building initiatives in the public health space and positively impact animal health systems.

## Methods

**Figure 1:**
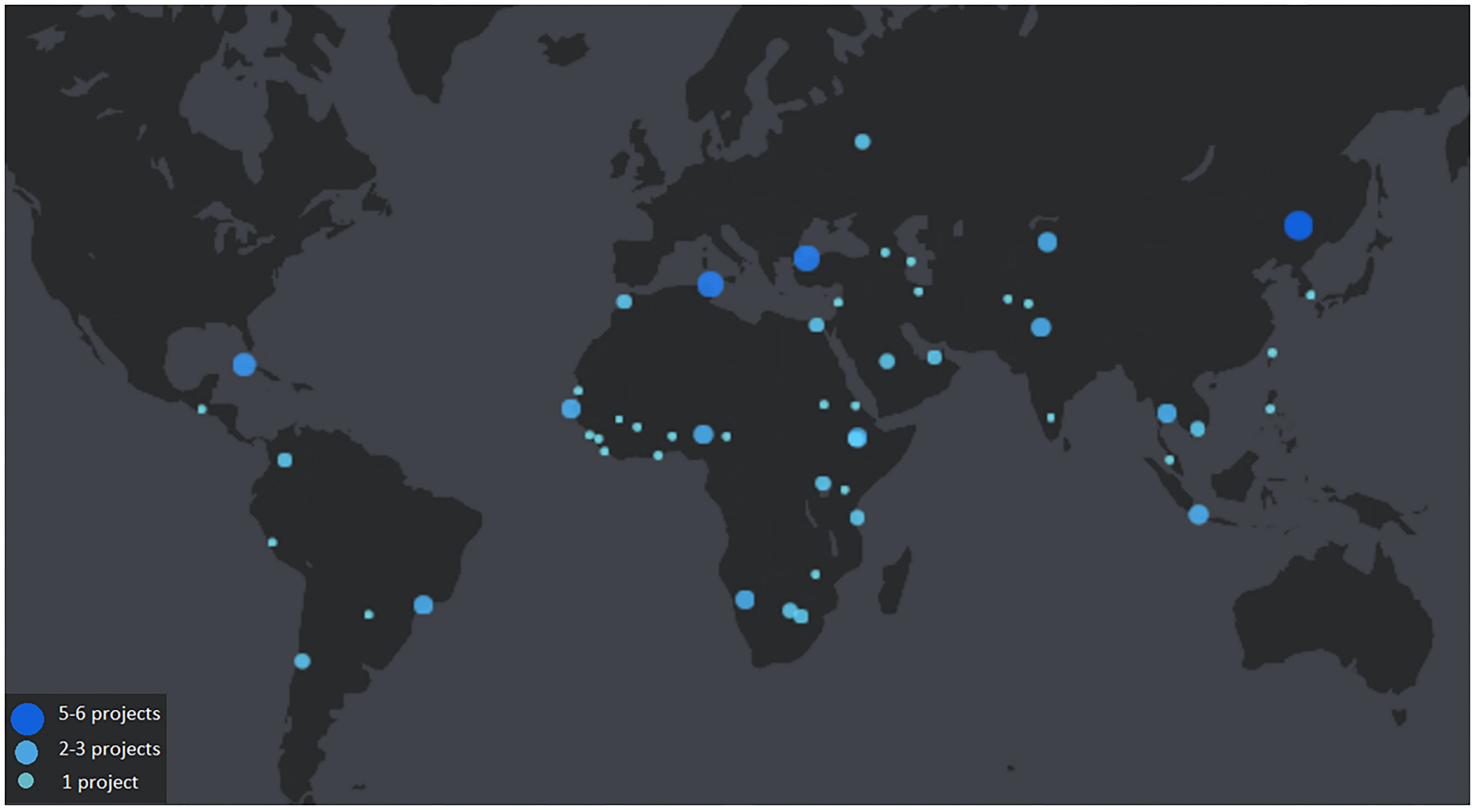
Global distribution of WOAH Laboratory Twinning projects implemented in the period 2006–2022. Larger dots indicate a higher number of projects implemented in one country.

**Figure 2:**
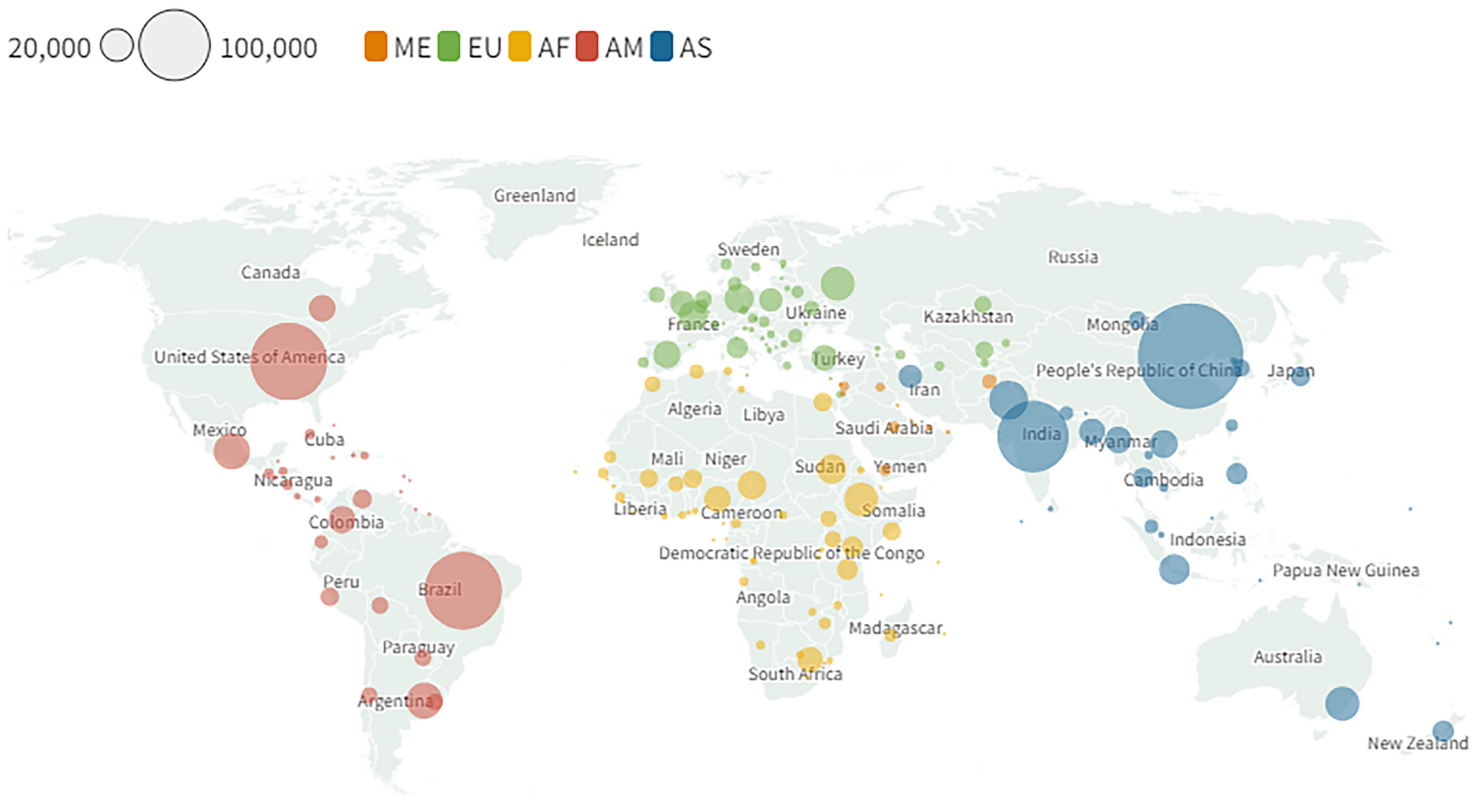
Global distribution of livestock biomass (including terrestrial and aquatic animals) in thousands of tons for the year 2018.

The data in this paper were sourced from a combination of public and internal WOAH records, in addition to a review of scientific literature. The map in Figure 1 was developed using the open access version of Flourish Studio (15) with historical data from project implementation publicly available in WOAH's website (16). The map in Figure 2 was created using the open access version of Flourish Studio with data from the animal biomass^1^ indicator developed by WOAH's Antimicrobial Resistance & Veterinary Products Department through a methodology adapted for the annual WOAH antimicrobial use data collection survey (17). The association between the origin of funding and the achievement of Reference Center status was tested using a Chi square test in MS Excel.

## Results

Between February 2008 and May 2022, 114 projects were initiated under the WOAH Laboratory Twinning Programme. Approximately two thirds (60%) of these projects have been completed, a third (30%) are underway, and 10% were canceled. The geographical location of the majority of twinnings, illustrated by Figure 1, matches the regions that were identified in 2006 as lacking laboratory expertise – Africa and Asia. African countries were the beneficiaries of 48 projects, Asia-Pacific received 29 twinnings, the Americas 16, Europe 12, and the Middle East only 7. It should nevertheless be noted that the Middle East region of WOAH is the least numerous in Member Countries (12) given its comparatively smaller geographical coverage in relation to the other four WOAH regions which have more Member Countries: Americas (31), Africa (54), Asia-Pacific (32), and Europe (53).

There is a great diversity of topics that interest twinning candidates. Although avian influenza and brucellosis were the most sought-after topics in the early years of the Programme, a cluster of new projects has favored the field of viral haemorrhagic fevers. Rabies has raised steady interest over time. Overall, Africa is the region with the highest percentage of donor-funded twinnings (98%), followed by the Americas (93%), and Europe (92%). The Asia-Pacific and the Middle East regions are the ones with the most self-funded projects^2^, at 81 and 33%, respectively. In all WOAH regions combined, 11.4% of twinning projects are self-funded.

Fifteen new WOAH Reference Centers have been designated as a direct result from participation in a WOAH laboratory twinning project. These Reference Centers are located in Abu Dhabi (1), Botswana (1), Brazil (1), Chile (1), China (4), India (1), Rep. of Korea (1), Russia (1), Senegal (1), Thailand (2), and Turkey (1) – totalling two new Reference Centers in Africa, two in the Americas, eight in Asia, two in Europe, and one in the Middle East. Out of the 15 projects that have resulted in new Reference Centers, only two were self-funded by the candidate countries. It was shown that there is no association between funding origin (self-funding vs. donor funding) and the achievement of Reference Center status. At present, the regions with the greatest number of WOAH Reference Centers are Europe, Asia - Pacific, and the Americas. There is a great disproportion in the distribution of Reference Centers inside these regions, favoring higher-income countries: the majority of Reference Centers in Europe are located in members of the European Union, and the bulk Reference Centers in Asia and the Pacific is in located in four countries: Australia, China, Japan, and the Republic of Korea; the vast majority of Reference Centers in the Americas are based in Argentina, Canada, and the USA.

The global livestock biomass – including terrestrial and aquatic animals – is unevenly distributed across the world. Four countries, China, Brazil, India, and the USA, have as much as 43% of the global livestock biomass, as shown in Figure 2, while in Africa and Europe the livestock biomass is more evenly distributed among neighboring countries.

## Discussion

Achievement of WOAH Reference Center designation is not a standard objective for every project. Aiming for such designation depends heavily on the starting capacity, staff commitment, equipment availability, and management engagement of the candidate institute, as WOAH twinnings are solidarity-based and no funds are spent on equipping and maintaining the laboratories. Nevertheless, at this point in the review of the Laboratory Twinning Programme, achievement of Reference Center designation is a concrete indicator of success, which is used to describe and compare progress in the regions involved in the Programme. The completion of the work plan set out in the beginning of the project as an indicator of success was considered. However, WOAH twinnings are not considered finalized until the work plan is fully implemented, which leaves all finished projects in a similar standing with regard to work plan implementation and is not a guarantee of success. That is why the review of the Twinning Programme includes the identification of success determinants and of indicators related to the sustainability of project outcomes.

Nearly half of the WOAH Laboratory Twinnings to date were implemented in Africa, with a number of African countries including Ethiopia, Namibia, Nigeria, Senegal, and Tunisia having benefitted from multiple twinnings. Nevertheless, this has not been translated into a higher ratio of new Reference Centers resulting from twinnings in the African region in relation to the other WOAH regions. This does not seem to match the original objective of the Programme, which was to even out the global distribution of veterinary laboratory expertise. There is a number of possible explanations for this finding: (1) the baseline capacity of the laboratories chosen to participate in the Programme may have been such that the improvement acquired during the project were not sufficient to elevate it to Reference Center level; (2) the laboratories may not have enough endogenous investment (as opposed to “donor investment”) or absorptive capacity to sustain and build on the work done during the project; or (3) the achievement of Reference Center status may not be a priority for many of the candidate countries, as this designation comes with its own set of bureaucracy, expenses, and responsibilities.

The origin of the funding supporting the projects has not shown to be associated the achievement of Reference Center status by the candidate laboratory. This is a reassuring finding given that nearly 90% of WOAH Laboratory Twinnings are donor funded. The location and topics of WOAH twinnings are significantly constrained by the availability of donor funding. Most commonly, these funds come with conditions related to the regions where the projects can be implemented and the topics that can be covered, according to donors' priorities and geopolitical interests. This is well illustrated by the low implementation of twinning projects in Central and South America, the high availability of funds for implementation of projects in Africa and Asia, and by the change in the popularity of certain twinning topics. In the early years of the programme, which included the 2009–2010 global swine/H1N1 flu outbreak, nearly all new projects focused on avian influenza. In the years after the Ebola crisis in West Africa, there was a significant increase in funding available for projects on viral haemorrhagic fevers. More recently, after African Swine fever started spreading in Asia, there was an uptick of funding for ASF. Wealthier countries with multiple participations in twinnings, such as China, seem to have the capacity to maintain interest and investment after the twinnings are concluded resulting in the systematic establishment of Reference Centers after twinnings.

Visual inspection of Figures 1, 2 seems to indicate that the distribution of WOAH Reference Centers and of Laboratory Twinning projects is not correlated with the global distribution of animal biomass. However, further qualitative analysis is needed to investigate the relationships between livestock distribution and animal health laboratory expertise. Nearly half of all WOAH Reference Centers are located in high-income countries in the European region, which has a relatively low concentration of animal biomass compared with China, Brazil, India, and the USA, the countries where the livestock biomass is the highest. It should be noted that the USA has a high number of WOAH Reference Centers in relation to other high-income countries, and that China, the country with the largest share of livestock biomass and the highest human population count, has taken significative advantage of the WOAH Twinning Programme, being the country that has the greatest number of new Reference Centers (4) resulting from laboratory twinnings. Interestingly, the Chinese example has also occurred in other emerging economies that belong to the BRICS, albeit at a smaller scale – Brazil, Russia and India have each one new Reference Center resulting from twinning projects. This seems to suggest that the ability of countries to take advantage of WOAH twinning projects and advance toward designation as a WOAH Reference Center is correlated to their income level and, thereby, with the capacity to leverage the investment made during the project possibly and take it forward with endogenous funds. This raises two questions: (1) What is the minimum standard that a candidate laboratory should have in order to be set up for success within the Programme; and (2) Which indicators other than Reference Center designation can be used to characterize successful projects.

Defining “success” is important to avoid settling for unstructured feedback describing successful twinning experiences. Given the solidarity-based character of WOAH twinnings, there is the risk that projects act as a band-aid for a bigger problem: the lack of investment in veterinary laboratories within public health networks. The minimum standard for candidate laboratories to benefit from a twinning project could be based on relevant sections of Chapter 1.1.1 of the WOAH Manual regarding Management of Veterinary Laboratories.

A monitoring and evaluation (M&E) tool for laboratory twinning projects is needed to better understand projects' impact, the sustainability of their outcomes in the medium to long term, and the characteristics shared by successful projects. Logically, the monitoring and evaluation components of the tools should be separated, as the indicators used to monitor projects' implementation would be different from those used to evaluate the project's outcomes, impact, and sustainability post-implementation. The process to create the M&E tool should build on the results of WOAH's recent work on laboratory sustainability (18), that analyzed data from a cohort of laboratories participating in the WOAH Performance of Veterinary Services Sustainable Laboratories (PVS Lab) missions and found that while capacity building efforts may have improved bench-top capacity in laboratories, this capacity is unsustainable. The M&E tool would not directly assess laboratory sustainability, as that is already covered by the PVS Lab tool, but rather integrate it into its indicators while focusing on the implementation and outcomes of laboratory twinning projects. Such tool would allow project implementors to learn from past experiences and better plan future projects, thereby promoting resource optimisation and improving the likelihood of success.

There is the opportunity to learn about the effectiveness of capacity building interventions in animal health laboratories from the data and experiences accumulated during the past 16 years. The factors associated with projects' success, failure and with the achievement of Reference Center status should be identified and systematized so as to ensure that resources allocated to the Programme are being well spent and that the Programme is complying with the objective of evening out the global distribution of veterinary laboratory expertise.

## Conclusion

WOAH's Laboratory Twinning Programme is a well-established and recognized initiative for capacity building in animal health laboratories. Its reach is global and the regions that received the most investment were the ones lacking laboratory expertise the most. However, this has not translated in a higher ratio of Reference Centers resulting from twinnings in these regions compared to the rest of the world. There is potential from learning from the experiences of the countries that have better leveraged their participation in the Programme, becoming Reference Centers in their own right. However, there are no data concerning the impact and the sustainability of the outcomes of twinning projects after their implementation is finished. An evaluation process that covers the outcomes and impact of the twinnings would help to optimize the implementation of future projects, ultimately providing better support to national veterinary services and improving animal health systems globally. A framework for such an evaluation is being developed and will be the subject of a future publication.

## Author contributions

MM conceived, designed, and performed the analysis, in addition to writing the paper. EA contributed to the analysis. MJ provided the biomass data. JR, WG, AN, and KH supervised the production of the paper. All authors contributed to the article and approved the submitted version.

## Funding

The work was supported by the World Organisation for Animal Health.

## Conflict of interest

The authors declare that the research was conducted in the absence of any commercial or financial relationships that could be construed as a potential conflict of interest.

## Publisher's note

All claims expressed in this article are solely those of the authors and do not necessarily represent those of their affiliated organizations, or those of the publisher, the editors and the reviewers. Any product that may be evaluated in this article, or claim that may be made by its manufacturer, is not guaranteed or endorsed by the publisher.

## Footnotes

^1^Biomass calculated includes the live animal population biomass.

^2^Self-funded projects are usually financed by the candidate country, which manages the project's budget independently from WOAH. In donor-funded projects WOAH's financial rules for laboratory twinnings apply.
